# Qualimap 2: advanced multi-sample quality control for high-throughput sequencing data

**DOI:** 10.1093/bioinformatics/btv566

**Published:** 2015-10-01

**Authors:** Konstantin Okonechnikov, Ana Conesa, Fernando García-Alcalde

**Affiliations:** 1 ^1^Department of Molecular Biology, Max Planck Institute for Infection Biology, D-10117, Berlin, Germany,; 2 ^2^Genomics of Gene Expression Lab, Centro de Investigación Príncipe Felipe, 46012, Valencia, Spain and; 3 ^3^Microbiology and Cell Science Department, Institute for Food and Agricultural Research, University of Florida at Gainesville, FL 32611-0700, USA

## Abstract

**Motivation:** Detection of random errors and systematic biases is a crucial step of a robust pipeline for processing high-throughput sequencing (HTS) data. Bioinformatics software tools capable of performing this task are available, either for general analysis of HTS data or targeted to a specific sequencing technology. However, most of the existing QC instruments only allow processing of one sample at a time.

**Results:** Qualimap 2 represents a next step in the QC analysis of HTS data. Along with comprehensive single-sample analysis of alignment data, it includes new modes that allow simultaneous processing and comparison of multiple samples. As with the first version, the new features are available via both graphical and command line interface. Additionally, it includes a large number of improvements proposed by the user community.

**Availability and implementation:** The implementation of the software along with documentation is freely available at http://www.qualimap.org.

**Contact:** meyer@mpiib-berlin.mpg.de

**Supplementary information:** Supplementary data are available at *Bioinformatics* online.

## 1 Introduction

High-throughput sequencing (HTS) is a powerful discovery method applied in genomics, transcriptomics and other omics disciplines. Projects such as ENCODE (Rosenbloom *et al.*, 2013) or BLUEPRINT (Adams *et al.*, 2012) generated terabytes of sequencing data, providing new insights into the molecular mechanisms of the cell. The sequencing technology itself has been improving continuously, allowing longer reads and deeper coverage at lower cost (Sims *et al.*, 2014). However, despite these advantages, HTS is prone to random errors and systematic biases, including polymerase chain reaction amplification problems, GC-content shift and read contamination (Ross *et al.*, 2013). To generate reliable conclusions from HTS data, these biases have to be detected and addressed accordingly. In this regard, several bioinformatics tools have been developed to perform quality control (QC) of the HTS data by analyzing raw reads and their derivatives in the form of sequencing alignments and other quantitative data (Patel and Mukesh, 2012).

In the context of large sequencing projects, it is also crucial to have a global overview of all samples in the experiment. Multi-sample analysis results comparison enables examination of data clustering and detection of possible outliers. There are special toolkits such as StatsDB (Ramirez-Gonzalez *et al.,* 2013) that allow creating detailed multi-sample analysis workflows; however, they require accurate construction of custom pipelines. Several existing NGS QC software tools including RNA-seq QC (DeLuca *et al.*, 2012) and RSeQC (Wang *et al.*, 2012) have only a few options for working with multiple samples. This is a major limitation, since sequencing experiments are often conducted using biological replicates and can include multiple conditions. Here, we present the second version of Qualimap (García-Alcalde *et al.,* 2012), a toolkit for QC of HTS alignment data. In Qualimap 2, we provide new analysis capabilities that allow multi-sample comparison of sequencing datasets. Additionally, we have added a novel mode for discovery of biases and problems specific to RNA-seq technology, redesigned the read counts QC mode and implemented numerous improvements.

## 2 Software description

Qualimap is a multiplatform user-friendly application with both graphical user and command line interfaces. It includes four analysis modes: *BAM QC*, *Counts QC*, *RNA-seq QC* and *Multi-sample BAM QC*. The latter two modes are introduced for the first time in version 2. Based on the selected type of analysis, users provide input data in the form of a BAM/SAM alignment, GTF/GFF/BED annotation and/or read counts table. The results of the QC analysis are presented as an interactive report within the graphical user interface, as a static report in HTML, as a PDF, or as a plain text file suitable for parsing and further processing. Typically, the report contains summary statistics of the dataset, description of input data, exploratory plots and histograms that visualize certain aspects of the data and help to detect potential problems.

One of the major new developments in Qualimap2 is the analysis mode called *Multi-sample BAM QC,* which allows combined QC estimation of multiple alignment files. For this purpose, Qualimap uses the metrics computed during the single-sample *BAM QC* procedure as input. The program loads the QC analysis results from each sample and creates a number of combined and normalized plots comparing specific properties. The types of generated plots correspond to single-sample *BAM QC* analysis plots. Analyzed samples can have different coverage depth, experiment type or even derive from different organisms.

**Fig. 1.**
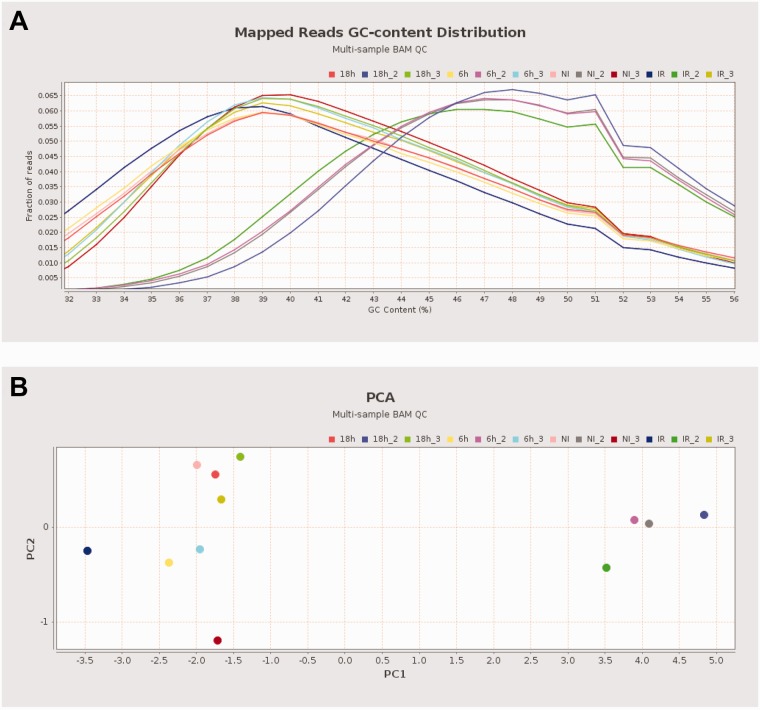
Multi-sample BAM QC analysis of a γH2AX ChiP-seq experiment in human cells comparing four different conditions (Koeppel *et al.*, 2015). The sequencing was performed in three batches. A single batch included samples in all conditions. (**A**) The GC-content distribution indicates a problem with the samples from the second batch. (**B**) The PCA biplot also demonstrates the second batch grouped together, despite different biological treatments

The simultaneous comparison of multiple samples allows examination of consistency between samples and visual detection of outliers (Fig. 1A). To estimate the variability between analyzed datasets, Qualimap performs a principal component analysis based on specific features derived from the alignment, including coverage, GC content, insert size and mapping quality (Fig. 1B).

Qualimap 2 also introduces a novel analysis mode called *RNA-seq QC.* This mode allows computation of metrics specific to RNA-seq data, including per-transcript coverage, junction sequence distribution, genomic localization of reads, 5′–3′ bias and consistency of the library protocol. A detailed comparison of Qualimap to RSeQC and RNA-seq QC tools that are focused on a similar goal can be found in Supplementary Table S1. The most significant difference to other tools is the subsequent RNA-seq QC analysis step that Qualimap performs after computation of read counts.

The mode *Counts QC* was completely redesigned to allow processing of multiple samples. Normally, this mode estimates the quality of the read counts that are derived from intersecting sequencing alignments within genomic features. Counts are usually applicable for analysis of differential gene expression from RNA-seq data (Anders *et al.,* 2013). Having multiple biological replicates per condition is common in RNA-seq experiments; therefore, it is beneficial to be able to analyze counts data from all generated datasets simultaneously. Multi-sample analysis of read counts allows inspection of sample grouping, as well as discovery of outliers and batch effects. Similar to the previous version, the *Counts QC* mode estimates the saturation of sequencing depth, read count densities, correlation of samples and distribution of counts among classes of selected features (Supplementary Figs. S1–S4). Additionally, new plots that explore the relationship between expression values and GC-content or transcript lengths are available for users. *Counts QC* is based on the NOIseq package for gene expression estimation (Tarazona *et al.*, 2012). The analysis results include a combined overview of the counts from all samples along with a QC report for each individual sample. Moreover, the analyzed datasets can have different conditions, e.g. treated and untreated. In this case, plots comparing groups of sample counts corresponding to particular conditions are generated (Supplementary Fig. S5).

## 3 Results and conclusion

Qualimap 2 is an application for exploratory analysis and QC of HTS alignment data written in Java and R. The major enhancement over the previous version lies in the ability to perform multi-sample analyses. Additionally, a large number of bug fixes and enhancements have been implemented since the initial release. An overview of novel features can be found in Table 1 and Supplementary Materials. In the present version, we have kept the concept of a simple, user-friendly application that follows an ‘open-source’ path. Qualimap 2 has gathered a community of users who frequently suggest new features and contribute their code. Notably, most of the novel features in *BAM QC* mode were proposed and tested by users. The public repository of Qualimap is hosted at *bitbucket.org/kokonech/qualimap*.

**Table 1.** Qualimap2—overview of novel features

Mode	Novel features and improvements
BAM QC	Advanced statistics of coverage, insert size, mismatch rate, etc.; duplicates extraction; homopolymer size control; performance and output data adaption
Multi-sample BAM QC	Comparison of coverage, GC-content, insert size etc. from multiple samples along with PCA-based summary
RNA-seq QC	Transcript coverage, 5′–3′ bias, alignment distribution, junction, strand-specificity analysis; counts computation
Counts QC	Multi-sample analysis (expression level, biotype, etc.) and condition comparison (expression level, GC bias, etc.)

## Supplementary Material

Supplementary Data
