# Supplementary material for: Qualimap 2: advanced multi-sample quality control for high-throughput sequencing data
Source: Bioinformatics. 2015 Oct 1;32(2):292–4. doi: 10.1093/bioinformatics/btv566 (PMC4708105; doi:10.1093/bioinformatics/btv566)
Supplement: Supplementary Data [file supp_32_2_292__index.html]

Qualimap 2: advanced multi-sample quality control for high-throughput sequencing data — Qualimap 2: advanced multi-sample quality control for high-throughput sequencing data — Supplementary Data 

# Qualimap 2: advanced multi-sample quality control for high-throughput sequencing data

## Supplementary Data

files

- Supplementary Data - docx file
