# Supplementary material for: Qualimap 2: advanced multi-sample quality control for high-throughput sequencing data
Source: Bioinformatics. 2015 Oct 1;32(2):292–4. doi: 10.1093/bioinformatics/btv566 (PMC4708105; doi:10.1093/bioinformatics/btv566)
Supplement: Supplementary Data [file supp_btv566_Qualimap2_supplementary_data_vs2_KO.docx]

*K. Okonechnikov et al.*

Qualimap 2: advanced multi-sample quality control for high-throughput sequencing data

**Supplementary Materials**

List of improvements in QualiMap v2 vs previous releases

**BAM QC mode**

- Added analysis of errors and mismatches
- Added additional metrics for coverage, mapping quality and insert size
- Added chromosome names in plots with chromosome limits
- Added option to exclude duplicate alignments during analysis
- Added option to set minimum homopolymer size
- Added option to provide SAM files as input
- Improved report in plain text format
- Improved warning reports (dataset analysis, memory control, etc.)
- Improved performance for long genomes
- Improved plots with chromosome name indication
- Multiple critical fixes including indel estimation, per-chromosome coverage computation, insert size limits, huge files support etc.

**Counts QC mode**

- Completely redesigned, added **support of multiple input** samples
- For global overview the following plots are available
  - *Counts log density*
  - *Scatterplot matrix*
  - *Sequencing saturation*
  - *Counts distribution*
  - *Features with low counts*
- Per-sample plots
  - *Sequencing saturation*
  - *Biotype detection*
  - *Counts per biotype*
  - *Length bias*
  - *GC bias*
- Comparison plots (comparing groups of samples under various conditions)
  - *Counts distribution*
  - *Features with low counts*
  - *Length bias*
  - *GC bias*
- Improved command line interface

**New mode: RNA-seq QC**

Analysis of metrics specific to RNA-seq technology

- Summary statistics: reads alignment, transcript coverage, junction sequence analysis
- Reads genomic origin: exonic, intronic or intergenic
- Transcript coverage (low, high, total)
- Transcript coverage histogram
- Junction analysis: known vs novel

**New mode: Multi-sample BAM QC**

Multi-sample comparison of BAM QC results

- Plots available from BAM QC showing all input samples simultaneously: *coverage across reference, coverage histogram, genome fraction coverage, duplication rate histogram, mapped reads GC-content distribution, clipping profile, mapping quality and insert size histograms*
- PCA of samples based on coverage, GC-content, insert size and mapping quality

**Compute counts tool**

- Added support for paired-end reads

A detailed explanation of analysis types and interpretation of output results can be found in the Qualimap user manual.

**Supplementary Table 1**

Comparison of existing RNA-seq quality control tools: **RSeQC** **v2.6**, **RNA-SeQC** **v1.1.8** and **Qualimap** **v2.1**. *Note: RSeQC and Qualimap also support general sequencing data analysis methods (including GC-content, number of mismatches and indels, insert size, mapping quality etc.). However, in this table these elements are not included, since it is focused on RNA-sequencing data analysis.*

| **Features and options** | **RSeQC** | **RNA-SeQC** | **Qualimap 2** |
| --- | --- | --- | --- |
| **General approach** | Command line execution only (Python scripts) | Execution either online via GenePattern or on command line | Launching possible from command line or GUI. |
| **Aligned reads**  **statistics** | Non-splice reads. | Total, unique, duplicate and alternative alignments.  Vendor Failed Reads. | Total, secondary, aligned to genes, non-unique, no-feature and ambiguous alignments. |
| **Read pairs statistics** | Pairs aligned, left/right | Pairs aligned, unpaired reads, base mismatch rate for each pair mate, chimeric pairs | Pairs aligned, left/right |
| **Strand-specificty detection** | Available | Available | Available |
| **Alignments location analysis** | Number of alignments in exons (5’UTR, 3’UTR, CDS), introns and TSS. | Exonic, intronic, intragenic, intergenic. | Exonic, intronic, intergenic |
| **Gene coverage analysis** | Gene coverage over gene body plot | Coverage gaps: count, length  Coverage plots | Coverage profile along genes (total, low, high),  coverage histogram. |
| **5’- 3’ bias**  **analysis** | - | Available | Available |
| **GC content**  **analysis** | Available | Available | Available |
| **Gene expression computation** | Available (RPKM) | Available  (read counts, RPKM) | Available  (read counts, RPKM) |
| **Expression profiling** | - | Efficiency (ratio of exon-derived reads to total reads sequenced)  rRNA reads | Detailed percentage of expressed exon type (protein-coding, pseudogene, lincRNA, rRNA, etc)  Low counts detection |
| **Multisample analysis and comparison** | Computes coverage of several samples together | Reports correlation between each sample pair | *In global mode:*  counts (coverage) density, scatterplot matrix, saturation, counts distribution.  *In comparison mode:*  between-sample counts distribution, low counts, biotype analysis , length bias, GC bias |

**Supplementary Figures**

Supplementary Figs. 1-5 were created by Qualimap 2.0 in Counts QC analysis mode. The input dataset was taken from a study by Weimer et al (2014); it consisted of 6 samples of mouse RNA-seq counts in 2 conditions: D-Glucosamine (GlcN) positive and negative. Each condition was presented by 3 biological replicates.

References

Weimer,S. et al. (2014) D-Glucosamine supplementation extends life span of nematodes and of ageing mice. *Nat. Commun*., 5, 3563.

**
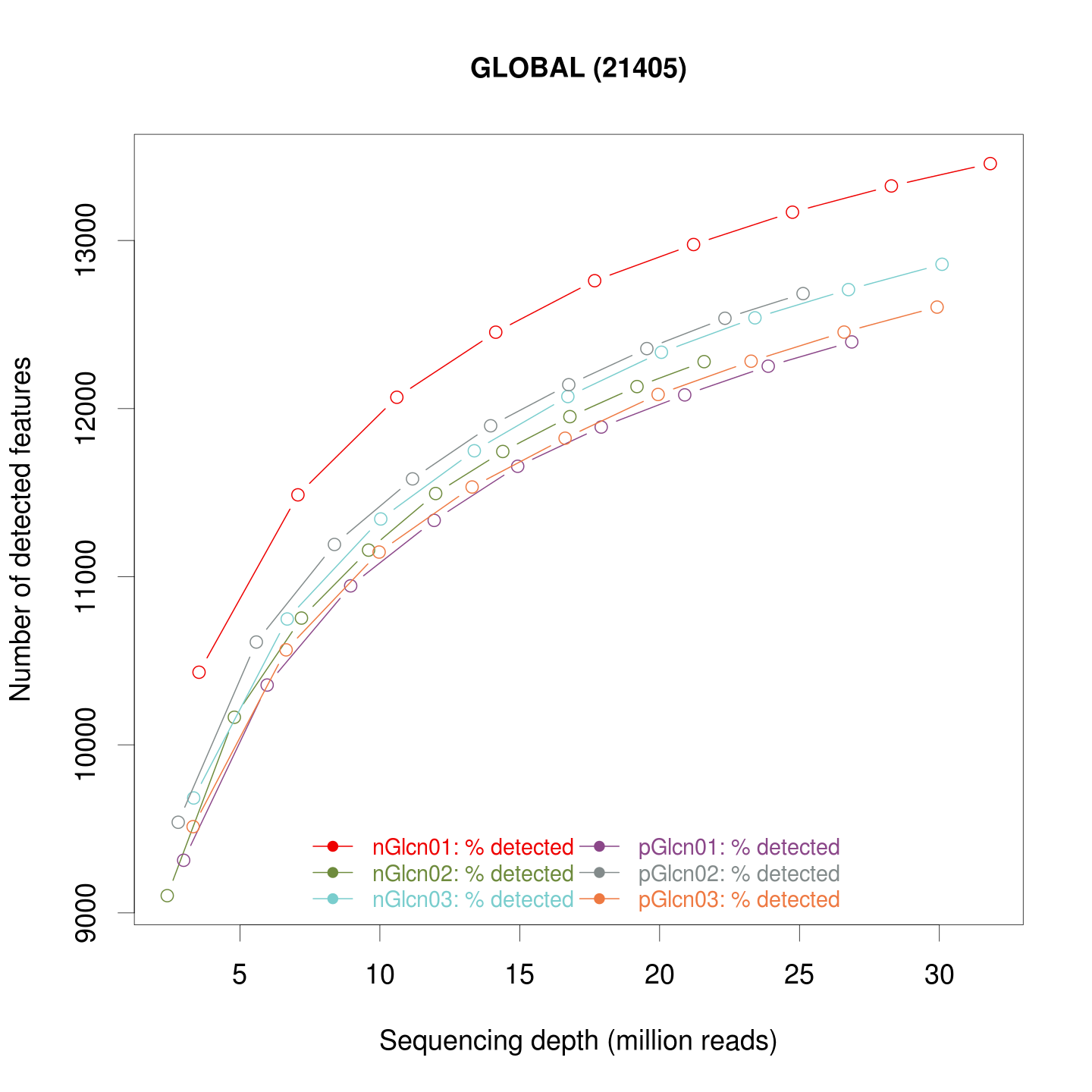
**

Supplementary Fig. 1. Global plot: sequencing saturation. This plot provides information about the level of saturation in the samples, helping users to decide if more sequencing is needed and if more features could be detected by increasing the number of reads.


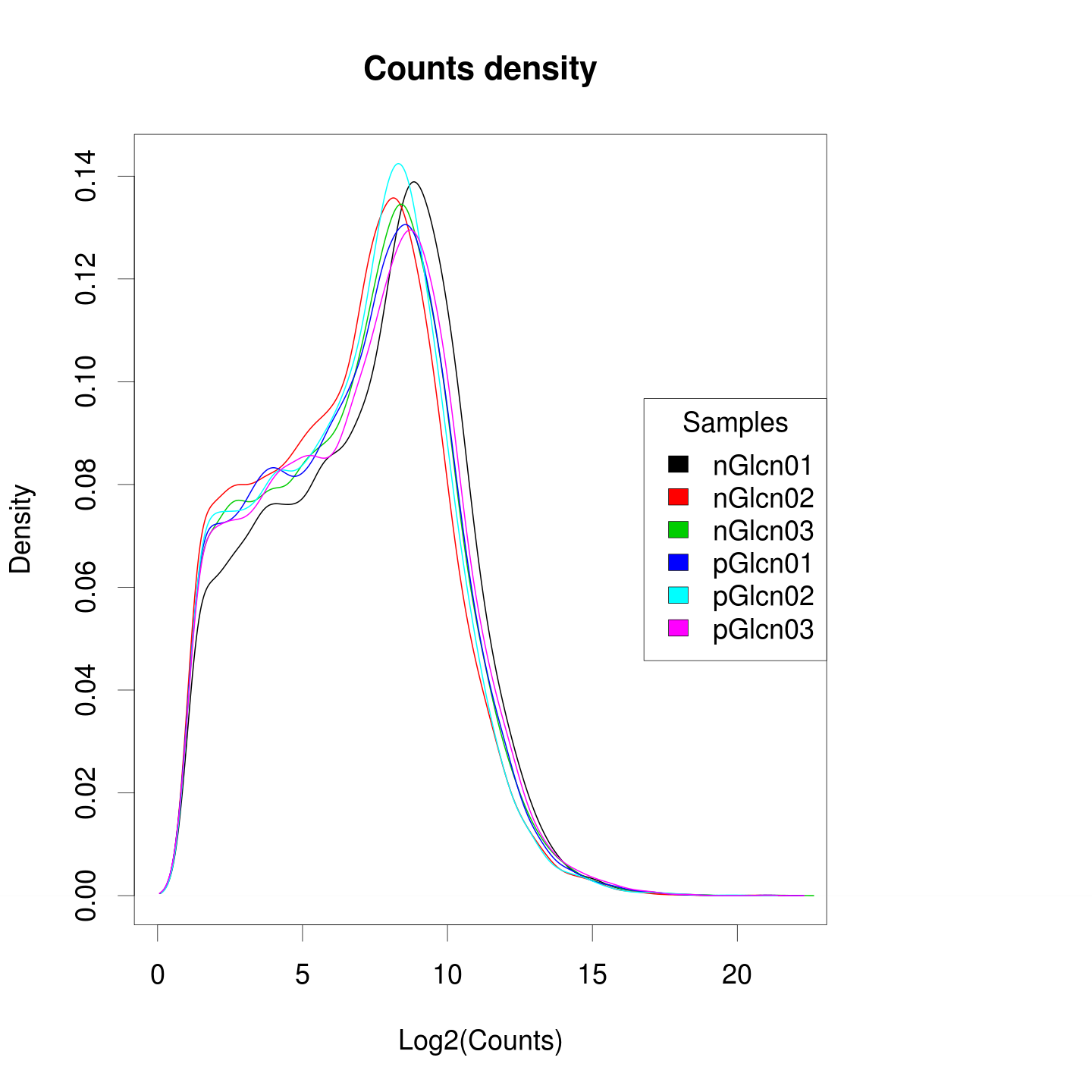


Supplementary Fig. 2. Global plot: counts density. This plot shows the density computed from the histogram of log-transformed counts. Comparison of density allows detection of outliers.


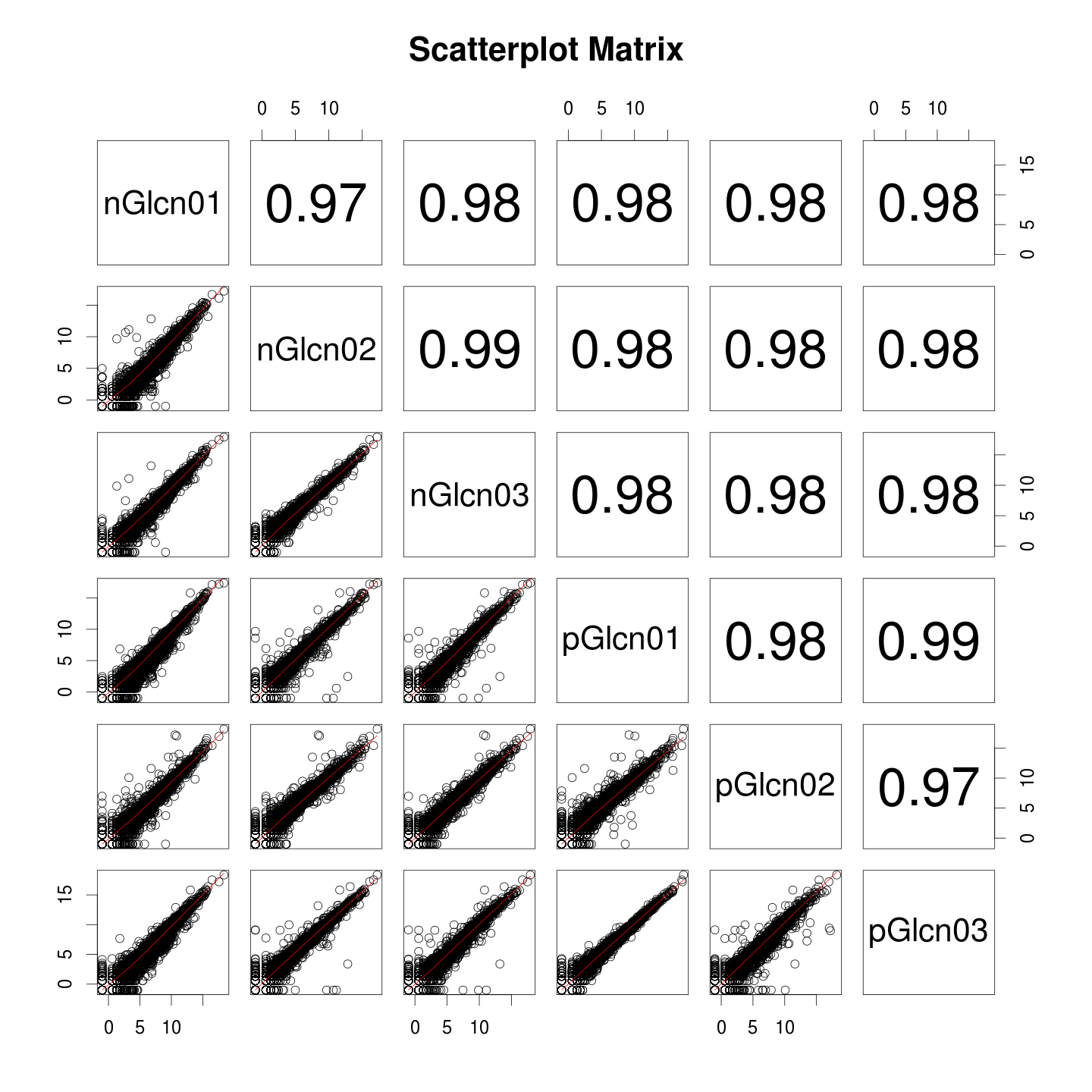


Supplementary Fig. 3. Global plot: scatterplot matrix. For each pair of samples the panel shows a scatterplot along with smoothed line (lower panel) and Pearson correlation coefficients (upper panel). Plots are generated using log-transformed counts. Such plots are helpful for comparison of difference between states.


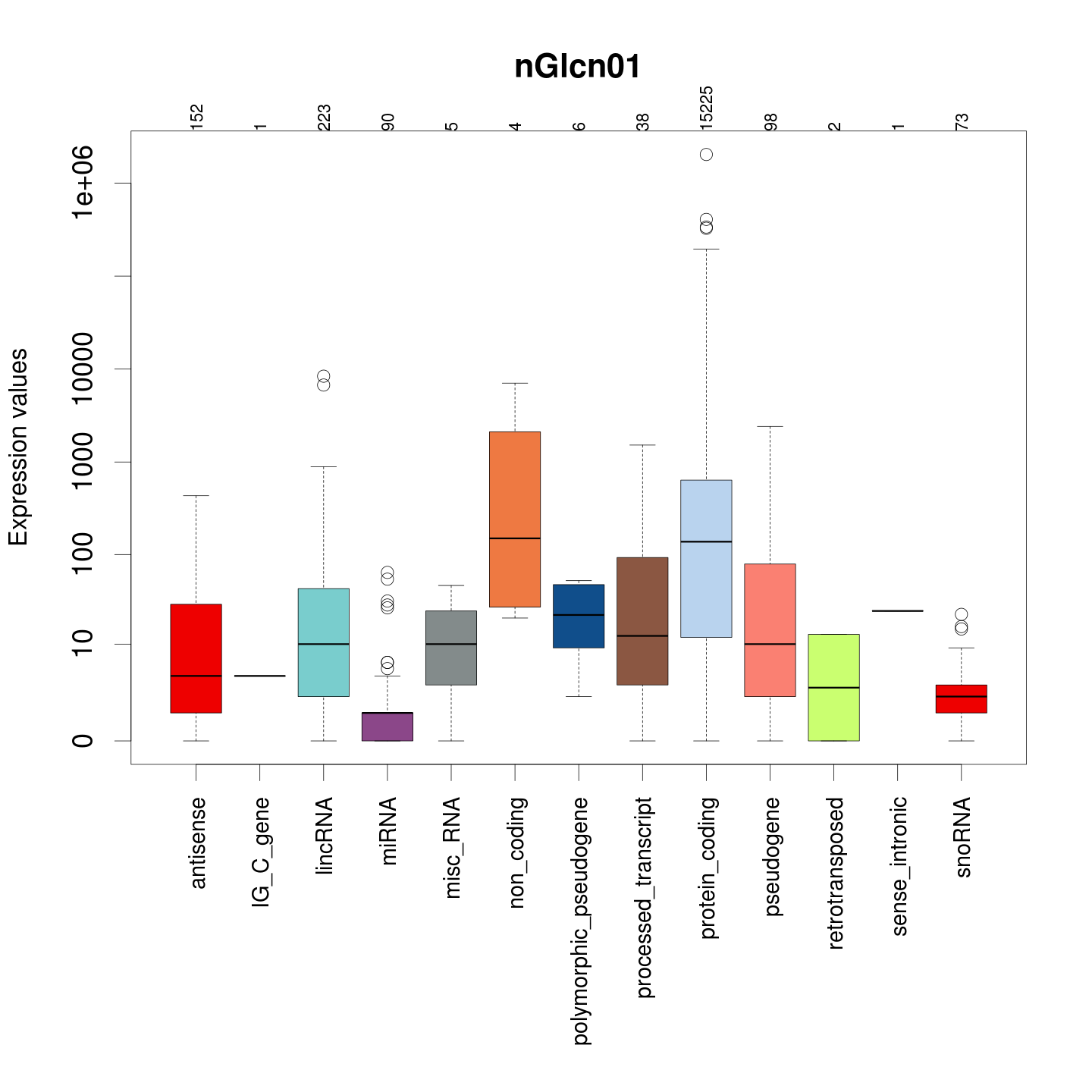


Supplementary Fig. 4. Single sample plot: counts per biotype. A boxplot describes the counts distribution among given biotypes of the features. This plot allows detecting whether a certain biotype doesn’t have enough data or by some reason over-increased. Note: heading indicates the name of the sample.


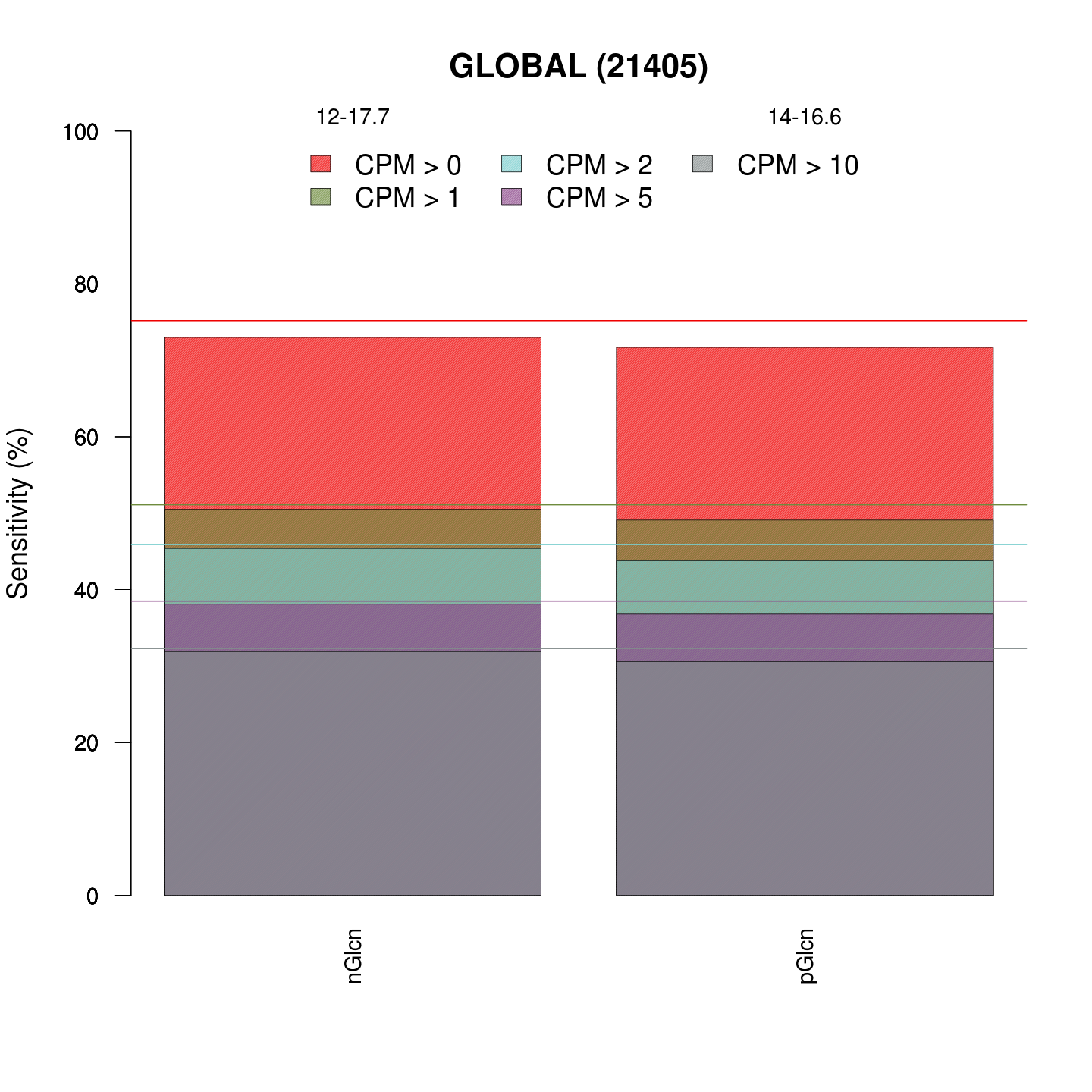


Supplementary Fig. 5. Comparison plot: features with low counts. This plot compares the proportions of features with more than 0, 1, 2, 5 and 10 Counts Per Million (CPM) across conditions. Additionally it allows detection of low-level expression profile of the dataset.
